# Vitamin D Level and Antibody Response to the BBIBP‐CorV Vaccine in Pregnant Women

**DOI:** 10.1155/jotm/1649421

**Published:** 2026-09-11

**Authors:** Shahab Falahi, Mahdi Vahabi, Hojjat Sayyadi, Azra Kenarkoohi

**Affiliations:** ^1^ Zoonotic Diseases Research Center, Ilam University of Medical Sciences, Ilam, Iran, medilam.ac.ir; ^2^ Faculty of Medicine, Ilam University of Medical Sciences, Ilam, Iran, medilam.ac.ir; ^3^ Non-Communicable Diseases Research Center, Ilam University of Medical Sciences, Ilam, Iran, medilam.ac.ir; ^4^ Department of Biostatistics, Faculty of Health, Ilam University of Medical Sciences, Ilam, Iran, medilam.ac.ir; ^5^ Department of Laboratory Sciences, School of Allied Medical Sciences, Ilam University of Medical Sciences, Ilam, Iran, medilam.ac.ir; ^6^ Department of Microbiology, Faculty of Medicine, Ilam University of Medical Sciences, Ilam, Iran, medilam.ac.ir

**Keywords:** antibody response, BBIBP-CorV vaccine, COVID-19, pregnant women, vaccine, vitamin D

## Abstract

**Background and Aims:**

Several factors influence the response to the vaccine, including age, gender, pregnancy, microbiota composition, immune function, sex hormones, and micronutrients (such as vitamin D). This study aimed to evaluate antibody responses induced by the BBIBP‐CorV vaccine in pregnant women and investigate its association with serum vitamin D levels.

**Methods:**

Serum samples were collected from 140 pregnant women 1–2 months after administering the second dose of the BBIBP‐CorV vaccine from August 2021 to April 2022. IgG antibody titers against RBD and serum vitamin D levels were measured using enzyme‐linked immunosorbent assay (ELISA). The relationship between vitamin D levels and anti‐RBD IgG antibody titer was investigated with linear regression analysis.

**Results:**

Anti‐RBD IgG antibodies were detected in 17% (24 out of 140) of the population studied. A positive correlation was observed between serum vitamin D level and anti‐RBD IgG antibody titer (*p* < 0.001).

**Conclusion:**

Our study showed that two doses of the BBIBP‐CorV vaccine elicited antibody responses against COVID‐19 in only a small proportion of pregnant women, suggesting that higher doses or alternative vaccine platforms may be required to protect pregnant women. Despite the existence of a relationship between vitamin D levels and antibody response in our study, prospective clinical trial studies are needed to draw definitive conclusions regarding the effect of vitamin D on antibody response.

## 1. Introduction

The COVID‐19 vaccines are an outstanding achievement in controlling the COVID‐19 infection that were developed quickly [1]. In most countries, high‐risk groups were vaccinated first [2, 3]. Factors such as age, gender, and the presence of underlying diseases were identified as risk factors for the severity of the COVID‐19 infection [4, 5]. Groups such as the elderly, healthcare workers, and pregnant women were considered high‐risk groups [4, 6, 7]. Pregnancy is associated with an increased risk of severe respiratory infections, and pregnant women are considered a high‐risk group for respiratory infections [8, 9]. During pregnancy, due to physiological, immunological, cardiopulmonary changes and hormonal fluctuations, morbidity and mortality from respiratory infections increase [8, 10]. Pregnant women are considered a high‐risk group for influenza infection [11], and this also applies to COVID‐19 [10]. Therefore, it is necessary to prioritize COVID‐19 vaccination in pregnant women; vaccines should be safe for both mother and fetus, and inactivated vaccines, such as the BBIBP‐CorV vaccine, can be a suitable platform for this population.

Maternal immunization is critical because, in addition to protecting the mother, the vaccine‐induced antibodies can be transferred to the fetus through the placenta (or through breast milk after birth), and maternal antibodies in the first months after birth are responsible for protecting the neonate against infections [11–13]. Pregnant women were not included in vaccine clinical trials, so the immunogenicity and effectiveness of vaccines in this group were not determined. During the COVID‐19 pandemic, micronutrients, including vitamin D, for strengthening the immune system received more attention [1]. The beneficial effects of vitamin D against respiratory infections and COVID‐19 have been mentioned in studies, and vitamin D supplementation has been recommended in several countries during the COVID‐19 pandemic. The vitamin D receptor is present in various immune cells, thereby affecting immune function, although the relationship between vitamin D and its effects on the immune system is complex [14, 15].

Vitamin D is a steroid hormone that affects the expression of more than 1000 genes, and most of these genes play a role in the innate and acquired immune systems. Vitamin D, by increasing the expression of antimicrobial peptides, inducing autophagy (for the clearance of intracellular pathogens) and apoptosis, increasing the xenophagic activity of macrophages, strengthening intercellular connections, and differentiating immune cells, plays a protective role against infections [14]. The vitamin D receptor and the enzyme CYP27B1 are present in immune cells, including T and B lymphocytes, macrophages, and dendritic cells. 1,25‐Dihydroxy vitamin D binds to VDR, then VDR binds to RXR (retinoid‐X receptor), and this heterodimer (VDR–RXR) binds to vitamin D–responsive elements on the target genes and, in this way, affects gene expression [14]. The target of most COVID‐19 vaccines is protein S or its RBD part, and studies have shown that antibodies against S or RBD can correlate with protection against COVID‐19. However, the protective threshold level for the COVID‐19 vaccines has not yet been determined. To the best of our knowledge, there is limited information on the effectiveness of the BBIBP‐CorV vaccine in pregnant women. Thus, the objective of the current study is to assess BBIBP‐CorV vaccine–induced antibody responses in a cohort of pregnant women in Ilam, Iran. In addition, some evidence suggests a positive effect of vitamin D on the response to vaccines [16, 17], while other studies do not [18]. Due to conflicting results, we examine the association between vitamin D levels and antibody titers after receiving two doses of the BBIBP‐CorV vaccine.

## 2. Materials and Methods

### 2.1. Study Design and Participants

This cross‐sectional study was conducted from August 2021 to April 2022 in Ilam, western Iran. The objectives of the study were explained to the participants, and their consent was obtained. This project was approved by the Ethics Committee of Ilam University of Medical Sciences (IR.MEDILAM.REC.1402.008). The ethical considerations in this study involved obtaining written informed consent from the research participants, ensuring confidentiality of participants’ information, and adhering to ethical principles in collecting library, research, and human information in accordance with the ethical principles of the Declaration of Helsinki. Participants were explicitly informed during the informed consent process about blood sample collection and the potential future use of their biological samples and research data.

Participants received two doses of the BBIBP‐CorV vaccine. The time interval between the first and second doses of the vaccine was 28 days. Inclusion criteria included participants’ willingness and consent to participate in the study, no obvious and symptomatic disease at the time of sampling (no suspicion of COVID‐19), no positive PCR test for COVID‐19 within 4 months before sampling, and no diseases such as diabetes, hypertension, or immunodeficiency. All participants in this study were Iranian pregnant women from Ilam province. Based on available information, the population under study was homogeneous in ethnic background and known physiological characteristics, and no significant differences were reported among ethnic groups. Serum samples from these individuals were collected 1–2 months after receiving the second dose of the BBIBP‐CorV vaccine and stored in a freezer at −80 C until testing was performed. For all 140 participating pregnant women, obstetric history information was extracted from medical records and from interviews with the mothers (Table 1). Accordingly, women were classified into four groups based on the number of previous deliveries: first delivery, second delivery, third delivery, and four or more deliveries. In women with two or more deliveries, the interval between the previous pregnancy and the onset of the next pregnancy (interpregnancy interval; IPI) was calculated in months and reported in three categories. These variables were considered to investigate the possible roles of cumulative burden and the interval between pregnancies in vitamin D status and anti‐RBD antibody response after receiving two doses of the BBIBP‐CorV vaccine.

**TABLE 1:** Obstetric history and interpregnancy interval of the study participants (*N* = 140).

Variable	Category/definition	*n*	%
Total participants		140	100.0

Parity (number of previous deliveries)	Primiparous (first delivery)	70	50.0
	Second delivery	49	35.0
	Third delivery	14	10.0
	≥ 4 deliveries	7	5.0

Women with ≥ 2 deliveries	(second, third, ≥ 4 deliveries)	70	50.0

History of abortion	No history of abortion	140	100.0
	≥ 1 previous abortion	0	0.0

History of the death of a live‐born child	No history	140	100.0
	≥ 1 previous death	0	0.0

Interpregnancy interval (IPI) among women with ≥ 2 deliveries (*n* = 70)	IPI < 24 months	4	5.7
	IPI 24–59 months	63	90.0
	IPI ≥ 60 months	3	4.3

### 2.2. Blood Sampling, Serum Preparation, and Preanalytical Standardization

Venous blood samples were collected from all participants using standard aseptic techniques in tubes containing gel/clot activator for serum preparation and then kept at room temperature for approximately 20–30 min to allow the clotting process to complete. All whole‐blood samples were processed on the same day as collection, and within 60 min of collection, the tubes were centrifuged at 3000 rpm for 6 min to separate the serum. The separated serum was immediately aliquoted into prelabeled polypropylene vials to avoid repeated freeze–thaw cycles. All aliquots were transferred to a −80°C freezer on the same day and stored in deep‐frozen conditions until testing. To ensure preanalytical stability, the same protocol was applied to all samples. These included avoiding prolonged storage at room temperature, minimizing the delay between blood collection and centrifugation, and transferring to the freezer on the same day.

Samples with severe hemolysis, significant lipemia, or other obvious changes were excluded from analysis, and subjects were resampled to avoid reducing the number of subjects studied. These measures were taken to reduce the risk of proteolytic degradation and analytical variability associated with storage and to improve the accuracy and reliability of 25(OH)D and anti‐RBD IgG antibody measurements. All tests were performed using a standardized protocol in accordance with the kit manufacturer’s instructions and internal quality control.

### 2.3. Laboratory Assay

The antibody titer against the RBD region in serum samples after Sinopharm (BBIBP‐CorV) vaccination was measured by ELISA using the Pishtaz kit (Pishtaz Teb Diagnostic, Iran, https://pishtazteb.com/). The test was performed according to the manufacturer’s instructions, with a cut‐off of 5.00 RU/mL. The samples with an antibody titer of less than 5.00 RU/mL were considered negative samples (seronegative), and those with an antibody titer above 5.00 RU/mL were considered positive samples (seropositive).

Vitamin D concentration in serum samples was measured using the ELISA method and the Pishtaz kit (Pishtaz Teb Diagnostic, Iran). Vitamin D concentrations, serum 25‐hydroxyvitamin D (25(OH)D), are considered to be the best biomarker for evaluating vitamin D status. Vitamin D concentrations measured according to the guidelines were placed in the following three groups: < 20.00 ng/mL as deficient, 30.00 < ng/mL as insufficient, and ≥  30.00 ng/mL as sufficient. The minimum detectable concentrations of specific IgG antibody against the SARS‐CoV‐2 RBD region and vitamin D in these kits are 0.10 RU/mL and 3.50 ng/mL, respectively. The sensitivity of the kit for measuring IgG antibodies against the SARS‐CoV‐2 RBD region is 97.1% with a 95% confidence interval.

The results of 25(OH)D concentration and anti‐RBD IgG antibody titer were reported based on the practical precision, limit of detection, and limit of quantification (LOQ) of the tests used. The cutoff value for distinguishing between sufficient and insufficient 25(OH)D levels, as well as the cutoff value for anti‐RBD IgG antibody positivity, was defined and reported strictly according to the kit manufacturer’s instructions.

### 2.4. Statistical Analysis

Antibody titer and vitamin D concentration data were presented as median. All statistical tests were performed using Stata version 11 (College Station, TX: StataCorp LP), and *p* < 0.05 was considered significant. A simple linear regression model was used to assess the relationship between the variables of the study.

To compare IgG titers between seropositive and seronegative women, as well as between different groups of vitamin D status (deficient, insufficient, sufficient), nonparametric tests appropriate for comparing two or more groups (e.g., Mann–Whitney *U* test for two groups and Kruskal–Wallis test for three groups) were used; the choice of test was based on the distribution of data and sample size in each subgroup. To investigate the role of gravida status (primigravida vs. multigravida) on vitamin D levels and IgG titers, these indices were compared in the two subgroups. To assess the potential role of maternal age in the relationship between vitamin D levels and anti‐RBD IgG titers, the correlation between gestational age and 25(OH)D concentration was calculated using an appropriate correlation coefficient (e.g., Spearman or Pearson, depending on the distribution of the data). In all tests, the statistical significance level was set at *p* < 0.05.

## 3. Results

**FIGURE 1:**
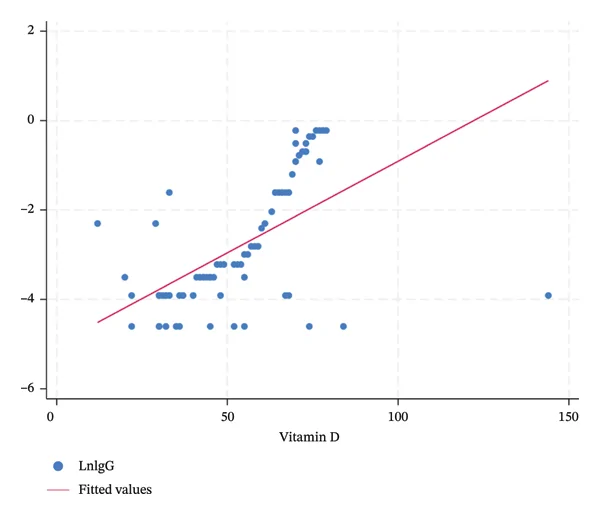
Simple linear regression model of the effect of vitamin D on antibody logarithm values (*R* ^2^ = 0.0990).

One hundred and forty pregnant women with a mean age of 30.7 (18–41, SD: 5.986191) years participated in our study. 130/140 (92.85%) women obtained the vaccine in the second trimester and 10/140 (7.14%%) in the third trimester. Serum samples were collected 1–2 months after the second dose of the BBIBP‐CorV vaccine. Anti‐RBD IgG antibodies were detected in 24 out of 140 (17.1%) participants (> 5.00 RU/mL). Geometric mean titers (GMTs) across all participants were approximately 0.70 RU/mL. The GMT for the positive group (IgG ≥ 5.00 RU/mL) was 15.22, and for the negative group (IgG < 5.00 RU/mL) was 0.13. None of the participants had a positive PCR test or a symptomatic infection of COVID‐19 before administering the vaccine until the time of sampling (self‐report). The vitamin D concentration of the serum samples was measured by the ELISA method and with the Pishtaz kit. 1.43% of the participants were in the vitamin D–deficient group, 2.86% in the insufficient group, and 95.71% in the sufficient group. The average vitamin D level in the study population was 52.51 ng/mL (range: 12–144 ng/mL). In contrast, the average vitamin D level in the seropositive group was 61.30 ng/mL, and in the seronegative group, it was 50.70 ng/mL (Table 2). Regression analysis showed a significant positive correlation between serum 25(OH)D levels and the titer of anti‐RBD IgG antibodies (*p* < 0.001), as shown in Figure 1. A one‐unit increase in vitamin D increases about 4.6% (95% CI = 0.02365–0.0691317) in the logarithm of anti‐RBD IgG antibodies. This finding suggests that vitamin D levels are significantly and positively associated with changes in anti‐RBD IgG titer. However, as this analysis was based on simple linear regression without adjustment for other covariates, this association should not be interpreted as independent of other potential factors.

**TABLE 2:** Descriptive statistics for the variables examined in the research.

Variable	Mean (SD) or *N* (%)	*p* value
Number of participants	140	NA
Mean age (years old)	30.7 (18–41)	NA
Gestational age at the time of receiving the first dose of vaccine		
Second trimester	130 (92.8%)	*p* > 0.05
Third trimester	10 (7.14%)	
Number of vaccine doses:		
Two doses	140 (100%)	NA
Underlying diseases:		
Yes	0 (0%)	NA
No	140 (100%)	
History of COVID‐19 infection:		
Yes	0 (0%)	NA
No	140 (100%)	
Antibody titer (RU/mL)	5.07	NA
Vitamin D level (ng/mL)	52.51	NA
Vitamin D level (ng/mL)		
Seropositive group	61.30	*p* < 0.001
Seronegative group	50.70	
Seropositivity rate (%)		
GMT	17.1	
Total participants	0.70	*p* < 0.001
Seronegative group	15.22	

*Note:* Comparison of vitamin D and GMT levels between the seropositive and seronegative groups was performed using the simple linear regression and Mann–Whitney *U* test. Other variables were descriptive statistics for the entire study population, and no intergroup comparisons were performed on them.

Abbreviation: NA, not applicable.

The obstetric characteristics of the participants are presented in Table 1. Of the 140 pregnant women, 70 (50%), 49 (35%), 14 (10%), and 7 (5%) were in their first, second, third, and fourth pregnancies, respectively, and none of the participants had a history of miscarriage or stillbirth. Among women who had two or more deliveries (*n* = 70), the interval between pregnancies was mostly in the range of 24–59 months (90%) (Table 1). No significant association was observed between the number of previous deliveries, IPI, and 25(OH)D levels or anti‐RBD antibody titers after vaccination (*p* > 0.05). These findings indicate that, in the study population, obstetric history and the interval between pregnancies showed no significant correlation with vitamin D status or antibody titers in unadjusted analyses. However, as a multivariable model was not applied, the possibility of residual confounding by these or other unmeasured variables cannot be excluded.

To investigate the possible role of gravida status in the immune response to the vaccine, 25(OH)D levels and anti‐RBD IgG titers were compared between the two groups of primigravida and multigravida. In this analysis, no significant or large differences were observed between the two groups in the distributions of vitamin D levels and IgG titers; in other words, gravida status did not appear to be a major determinant of the antibody response to the BBIBP‐CorV vaccine in this population. Comparative graphs of these two groups are presented in the corresponding figures.

To assess the potential role of maternal age as a confounder in the relationship between vitamin D and anti‐RBD IgG titers, the correlation between maternal age and 25(OH)D levels was examined. In this analysis, gestational age showed no significant correlation with maternal vitamin D status in this dataset, suggesting it is less likely to have substantially confounded the observed association between vitamin D status and anti‐RBD IgG titers; however, this could not be formally tested in a multivariable model. The scatterplot of this relationship is shown in the corresponding figure.

**FIGURE 2:**
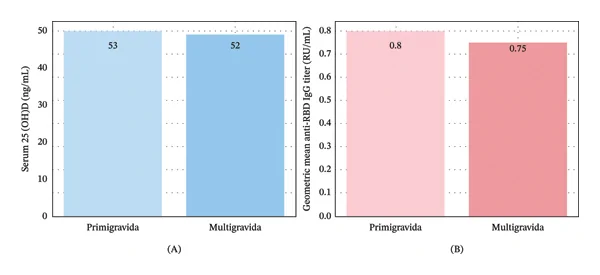
Vitamin D and IgG by gravida status (primigravida vs multigravida): Figure legend: (A) serum 25(OH)D levels (ng/mL) in primigravida and multigravida pregnant women. (B) Anti‐RBD IgG titers (RU/mL) in the same two groups.

These additional analyses show that, although the rate of anti‐RBD IgG seropositivity after BBIBP‐CorV vaccination in pregnant women is relatively low (17%), vitamin D levels are positively and significantly associated with antibody titers. Unadjusted analyses showed no significant correlation between maternal age or gravida status and vitamin D levels, suggesting these variables are less likely to confound this association substantially; however, because multivariable adjustment was not performed, this relationship cannot be described as independent of maternal age and gravida status (Figure 2).

**FIGURE 3:**
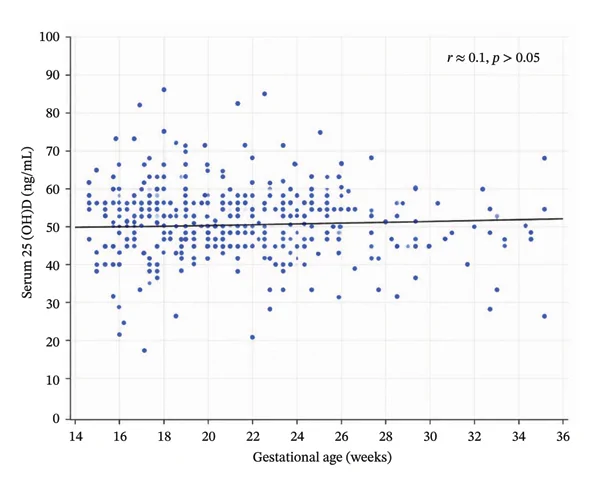
Relationship between maternal age and serum vitamin D levels in BBIBP‐CorV–vaccinated pregnant women.

Evaluation of gestational age at the time of blood sampling showed that serum 25(OH)D concentrations did not differ significantly across gestational ages in the second and third trimesters. The scatter plot and linear regression analysis revealed no statistically significant or clinically relevant correlation between gestational age (weeks of pregnancy) and vitamin D levels Figure 3.

All pregnant women participating in this study were Iranian residents of Ilam province, and no significant variation in ethnicity, race, or skin color was reported; therefore, separate analyses by ethnic subgroup were not possible.

## 4. Discussion

The findings of our study showed that the seropositivity rate after the BBIBP‐CorV vaccine in pregnant women is low. Our results also showed an association between serum vitamin D levels and anti‐RBD IgG antibody titer. The results of our study indicated that the seropositivity rate was lower than in other studies [16, 17]. The seropositivity rate after BBIBP‐CorV vaccine varies between studies, possibly due to differences in the type of kits used, the type of antibody measured, the time interval between the last vaccine dose and blood sampling (timing of antibody measurements), the number of vaccine doses, demographic characteristics, subjects’ age, diet, micronutrient status, specific conditions such as pregnancy, and even which trimester of pregnancy they were in.

Our results are not consistent with previous studies on COVID‐19 vaccination during pregnancy [16, 17]. For example, in a study in Sri Lanka, a high rate of seroconversion was reported in pregnant women after administration of the BBIBP‐CorV vaccine. In the Sri Lankan study, the presence of total antibodies (IgM, IgG, or IgA) against the receptor‐binding domain (RBD) of SARS‐CoV‐2 was measured. However, in our study, only IgG antibodies against the RBD of SARS‐CoV‐2 were measured, which may have affected the seropositivity rate [16].

A possible explanation for the reduced seropositivity rate observed in our research (17%), compared with other studies, could be variations in the timing of vaccination relative to gestational age. Our data showed that gestational age does not appear to influence the maternal vitamin D status and is unlikely to confound the observed association between vitamin D levels and the humoral response (anti‐RBD IgG titers) to the BBIBP‐CorV vaccine.

In our group, a majority of individuals (90%) were vaccinated during the second trimester, while merely 10% received the vaccine in the third trimester. The immune system during pregnancy is largely tolerogenic and associated with reduced inflammatory responses, especially in the last months of pregnancy [18]. In other words, the immune response changes throughout the various trimesters of pregnancy, which could influence the immune responses elicited by vaccination [18, 19]. Consequently, differences in the timing of vaccination during pregnancy (i.e., the trimester in which women received the vaccine) across studies may partly explain differences in seropositivity rates.

Another important factor that may contribute to the different seropositivity rates across studies is the timing of antibody measurements. In our study, antibody levels were assessed 1–2 months after the second dose of vaccine, whereas several other studies measured antibody responses at earlier time points [16]. It is well established that antibody titers decline with time after vaccination, which may be partly responsible for our low seropositivity rate.

Antibody responses following the BBIBP‐CorV vaccine have also been studied in other populations: in one study, a 31.1% seroconversion was reported after two doses of the BBIBP‐CorV vaccine in hemodialysis patients [20]. In another study, antibodies were detected in 55.6% of patients with hemoglobinopathies in the study population 1 month after the second dose of the BBIBP‐CorV vaccine [21]. In these populations, the seropositivity rate following the BBIBP‐CorV vaccine is also not favorable.

Our results also showed an association between serum vitamin D levels and anti‐RBD IgG antibody titers following the BBIBP‐CorV vaccine. There is evidence that vitamin D supplementation plays a role in preventing respiratory tract infections, especially in communities where vitamin D deficiency is common [22]. A study reported that vitamin D deficiency is associated with increased morbidity/mortality from COVID‐19 infection [23]. Available data have shown that adequate levels of vitamin D (≥ 30.oo ng/mL) are associated with better outcomes of COVID‐19 infection [24]. Although the available evidence indicates the beneficial effect of vitamin D against respiratory infections [24], the exact effect of vitamin D on the immunogenicity and efficacy of vaccines is still unclear.

Animal studies support the beneficial effect of vitamin D on vaccine response. According to the results of an animal study, 1,25(OH)2VitD at the vaccination site causes DC to migrate to lymphoid organs, where they stimulate T and B cells to produce a potent immune response to diphtheria vaccination. 1,25(OH)2VitD boosts the production of specific antibodies when given to mice along with the influenza vaccine [25].

Previous studies on the effect of vitamin D on vaccine response have mixed results. A study suggests that vitamin D deficiency during hepatitis B vaccination is associated with a weaker immune response [26]. Similarly, in patients with chronic kidney disease, a lack of vitamin D is linked to a weak response to the hepatitis B vaccine [27]. In contrast, a group of researchers reported that low vitamin D levels in subjects were associated with higher antibody titers after papillomavirus vaccination [28]. In a clinical trial conducted in older adults with vitamin D deficiency, vitamin D supplementation did not affect the antibody response to the influenza vaccine [29].

Regarding the COVID‐19 vaccines, a study of 101 healthcare workers 6 months after the second dose of the BNT162b2 vaccine found a significant correlation between baseline 25OHD levels and antibody responses [30]. Similarly, another study was conducted on 97 HCWs who received the first dose of the SARS‐CoV‐2 BNT162b2 vaccine. Vitamin D concentrations were measured at baseline, and antibody responses were measured at multiple time points. Results showed that serum vitamin D levels greatly influenced response to the vaccine [31].

In contrary, in a study conducted 3 months after the second dose of the BNT162b2 vaccine on 712 individuals, no clear correlation between vitamin D levels and the antibody titer was seen [32]. Both antibody titers and the risk of SARS‐CoV‐2 breakthrough infection in vaccinated individuals were unaffected by vitamin D supplementation [33]. In another study, no significant correlation was found between vitamin D levels and antibody responses following an inactivated COVID‐19 vaccine [34].

In addition to individual and environmental factors that can influence vitamin D status and the immune response to vaccines, genetic and demographic differences may also influence serum 25(OH)D levels and, consequently, the immune response. These mechanisms may explain part of the observed associations between vitamin D status and antibody response after vaccination in some studies [1, 2]. On the other hand, vitamin D status across populations is influenced by factors such as sunlight exposure, skin pigmentation, dietary patterns, supplementation, and demographic background, including skin phototypes. Also, differences in 25(OH)D levels between populations and even between ethnic groups have been reported in various studies, suggesting that demographic background may be one of the factors affecting vitamin D status. However, in the present study, all participants were Iranian and residents of Ilam province; therefore, the study population was relatively homogeneous in terms of geographical and ethnic backgrounds. This homogeneity can reduce the possibility of confounding effects due to ethnic differences, but at the same time limits the generalizability of the results to more ethnically diverse populations [35–38].

This study has several limitations that should be acknowledged: baseline anti‐RBD antibody levels were not assessed before vaccination; thus, pre‐existing immunity from prior symptomatic or asymptomatic SARS‐CoV‐2 infection could not be completely excluded. Although participants reported no history of clinically suspected COVID‐19 and no prior positive PCR test, undetected asymptomatic infection remains possible. Furthermore, given the use of a whole‐virus inactivated vaccine (BBIBP‐CorV), anti‐nucleocapsid (NP) IgG antibodies cannot reliably distinguish between vaccine‐induced and infection‐induced immunity. These factors should also be considered when interpreting the results.

In addition, baseline serum vitamin D levels were not measured before vaccination, limiting our ability to determine the temporal relationship or causality between vitamin D levels and the antibody response. Given that both vitamin D concentrations and antibody levels were measured at the same time, 1–2 months following the second dose of the vaccine, the results indicate a correlation instead of a cause‐and‐effect link. It is important to note that the study group comprised pregnant women, who typically receive routine vitamin D supplementation during prenatal care, which may lead to a relatively stable vitamin D level in the short term. However, this highly skewed distribution of vitamin D status, with the vast majority of participants classified as sufficient, represents a significant methodological limitation. The near‐absence of participants with insufficient or deficient vitamin D levels substantially limits the statistical power to detect meaningful differences in antibody response across vitamin D status categories. It limits the generalizability of the findings to populations with broader variation in vitamin D levels. The predominance of vitamin D sufficiency in this cohort, likely attributable to routine prenatal supplementation, may have created a ceiling effect, potentially attenuating the true magnitude of the association between vitamin D status and vaccine immunogenicity.

Furthermore, the potential influence of confounding variables, including maternal age, gravida status, gestational age, and obstetric history, was examined only through unadjusted bivariate analyses rather than a multivariable regression model. Although no statistically significant correlations were identified between these variables and vitamin D levels in bivariate testing, formal adjustment through a multivariable framework was not performed. Therefore, all reported associations should be interpreted as unadjusted, and the possibility of residual confounding by these or other unmeasured variables, such as dietary habits, sun exposure, socioeconomic status, or comorbidities, cannot be excluded.

The relatively low seropositivity rate observed in this study warrants careful interpretation. Several factors may have contributed to this finding, including the immunomodulatory effects of pregnancy on humoral immune responses, the timing of antibody measurement 1–2 months postvaccination (which may not represent peak antibody titers for all participants), and potential variability in cold‐chain integrity or vaccine lot characteristics inherent to inactivated whole‐virus platforms such as BBIBP‐CorV. Additionally, as previously noted, the possibility of undetected prior SARS‐CoV‐2 infection cannot be entirely excluded; conversely, some participants with apparent seronegativity may have mounted cellular immune responses not captured by anti‐RBD IgG measurement alone. These considerations should be taken into account when comparing seropositivity rates with those reported for mRNA‐based vaccines in nonpregnant populations. Nevertheless, further prospective longitudinal research with measurements taken before vaccination and serial assessment of immune responses is needed to enhance our understanding of causation and the timing of effects.

## 5. Conclusion

In conclusion, our study showed that two doses of the BBIBP‐CorV vaccine induced antibody responses against COVID‐19 in a small proportion of pregnant women, suggesting that higher doses or alternative vaccine platforms (such as mRNA COVID‐19 vaccines) may be required to protect pregnant women. In addition, our study found that vitamin D levels were associated with antibody levels. However, to draw definitive conclusions about the effect of vitamin D on vaccine‐induced antibody responses, future studies should be oriented towards prospective clinical trial designs.

## Author Contributions

The authors confirm contribution to the paper as follows: conceptualization and writing–original draft (lead): **Azra Kenarkoohi** and **Shahab Falahi**. Laboratory test: **Shahab Falahi**, **Azra Kenarkoohi**, and **Mahdi Vahabi**. Statistical analysis: **Hojjat Sayyadi**.

## Funding

This work was supported by the Ilam University of Medical Sciences (14021004/15).

## Disclosure

All authors reviewed the results and approved the final version of the manuscript.

## Conflicts of Interest

The authors declare no conflicts of interest.

## Data Availability

The data that support the findings of this study are available on request from the corresponding author. The data are not publicly available due to privacy or ethical restrictions.
